# Equitable Aging Among Migrants: A Concept Analysis and Model Development for Transcultural Nursing Care

**DOI:** 10.1177/10436596251359129

**Published:** 2025-08-12

**Authors:** Areej Al-Hamad, Yasin M. Yasin, Sepali Guruge, Kateryna Metersky, Lu Wang, Cristina Catallo, Hasina Amanzai, Zhixi Cecilia Zhuang, Rezwana Rahman, Andy Zhang

**Affiliations:** 1 Toronto Metropolitan University, Ontario, Canada; 2 University of New Brunswick, Canada

**Keywords:** equitable aging, older migrants, transcultural nursing, culturally responsive care, concept analysis

## Abstract

**Introduction:** Older migrants often face systemic barriers such as limited access to health care, social support, and culturally appropriate services, which hinder dignified aging. This concept analysis aims to define equitable aging among migrants and develop a model to guide transcultural nursing care. **Methodology:** Using Walker and Avant’s concept analysis method, a systematic search following PRISMA-ScR guidelines yielded 351 records. After deduplication, 349 titles and abstracts were screened, 138 full-text articles were reviewed, and 68 studies were included in the final analysis. **Results:** Three defining attributes of equitable aging were identified: fair and just provision of health and social care; elimination of systemic barriers; and inclusive culturally responsive care. A conceptual model was developed, aligning equitable aging with key principles of transcultural nursing. **Discussion:** This concept analysis offers greater conceptual clarity on equitable aging among migrants and identifies defining attributes that may inform future development of theoretical models and culturally responsive practices.

## Introduction

Global demographic shifts have made aging a major concern for policymakers and practitioners (Bengtson, 2018). By 2050, the population aged 60 and above is expected to reach nearly two billion (World Health Organization [WHO], 2024), necessitating inclusive frameworks that support diverse aging experiences (Sen & Nagaram, 2024; Settersten & Angel, 2011). Equitable aging refers to the ability of older migrants to age with dignity, access to culturally appropriate care, and the opportunity to thrive regardless of their migration history, language, or socioeconomic status (American Association of Retired Persons [AARP] International, 2022). As global migration continues to rise, ensuring equity in aging for diverse migrant populations has become a pressing public health and social concern (Ayón et al., 2020). Migrants often face intersecting barriers including language difficulties, limited health care access, cultural misunderstandings, and systemic discrimination that compromise their ability to age well in host countries (Ayón et al., 2020; De Voogd et al., 2020; Tang et al., 2020). Despite growing attention to health equity and aging, there remains limited conceptual clarity on what equitable aging entails in the context of migration.

In the literature, definitions of equitable aging often emphasize structural determinants of health, fairness in resource allocation, and inclusive health systems, yet they rarely account for the distinct experiences of aging migrants (Carlsson, 2023). Migrants, particularly those with precarious legal status or from marginalized groups, face compounded disadvantages such as limited access to culturally appropriate care, economic insecurity, and social exclusion (Ayón et al., 2020; Siddiq et al., 2023). Equitable aging emphasizes fairness in access to health care, social support, and economic security (AARP International, 2022), particularly for migrants, who face lifelong barriers such as language challenges, legal insecurity, and socioeconomic disadvantages (Ayón et al., 2020; De Voogd et al., 2020; Tang et al., 2020). Migrant aging experiences are shaped by intersecting factors such as legal status and gender. Refugees frequently contend with trauma and health issues linked to forced displacement (Siddiq et al., 2023), while labor migrants often grow older with limited financial security due to prolonged engagement in low-wage employment (Szabó & Goodin, 2024). Undocumented migrants face heightened risks as they age, largely because of restricted access to health care and social services (Ayón et al., 2020). Migrant women, in particular, experience layered vulnerabilities driven by both gender and legal precarity (Carlsson, 2023; Kc et al., 2024).

Understanding equitable aging requires integrating social determinants of health, as older migrants’ outcomes are shaped by lifetime access to education, income, and health care not just individual choices (Hooten et al., 2022). Structural inequities, including language barriers and limited access to culturally responsive services, worsen late-life health outcomes (Lebrun & Shi, 2011; S. Lin, 2024). Multiple social identities including age, ethnicity, gender, and immigration status compound these disparities, calling for tailored policy responses (Hossen et al., 2023; Peckham et al., 2018). Addressing these inequities involves expanding health care access, income support, and culturally competent services (Kobayashi & Khan, 2023; Ro et al., 2022). Translation services, outreach programs, and diverse staffing improve health literacy and care navigation (Siddiq et al., 2023). Community-based solutions, such as faith groups, caregiver support, and senior associations, strengthen social ties and reduce isolation (Jacobsen et al., 2023; Qi et al., 2022). Coordinated policy and advocacy are essential to integrate aging and immigration strategies, enforce anti-discrimination laws, and improve data collection (Georgeou et al., 2023).

Transcultural nursing plays a key role in promoting equitable aging. Nurses must integrate patients’ cultural beliefs, health practices, and language needs into care delivery (Tosun et al., 2021). Tailored interventions such as interpreter use, traditional healing inclusion, and trust-building enhance care accessibility (Henly, 2016; Prosen et al., 2021). Leininger’s framework of culturally congruent care emphasizes cultural preservation, accommodation, and repatterning (Leininger, 2002), aligning with Im and Lee’s (2018) call for evidence-based, culturally competent models to improve migrant health outcomes.

Despite growing research on aging, the concept of equitable aging remains inconsistently defined, especially for migrant populations (AARP International, 2022). Existing theories often overlook intersecting disadvantages such as restrictive legal systems and cultural mismatches in care (Glass & Vander Plaats, 2013; Hossen et al., 2023; S. Lin & Fang, 2023). Achieving equitable aging requires bridging transcultural care, policy, and social determinants. Achieving equitable aging for older migrants require understanding how transcultural health care, policy, accessibility, and social determinants intersect. More consistent terminology is essential to align academic discourse, policy frameworks, and community interventions with older migrants’ health needs. Integrating transcultural health strategies into aging policies can bridge disparities, promote inclusivity, and foster culturally congruent care.

To clarify the conceptual focus of this article, we intentionally use the term “equitable aging among migrants” to position equitable aging as the central concept, with migration serving as the contextual lens through which it is explored. While “equitable aging” broadly refers to the fair distribution of resources, opportunities, and outcomes in later life (AARP International, 2022), its expression among migrants is uniquely shaped by structural inequities, cultural dislocation, and policy-level barriers. Migrant older adults often face intersecting forms of exclusion, including limited access to linguistically and culturally appropriate services, precarious legal status, and social isolation (Hossen et al., 2023; Peckham et al., 2018). By framing the analysis in this way, the concept of equitable aging is situated within a sociopolitical context that recognizes how migration experiences influence individuals’ ability to age with dignity, autonomy, and inclusion.

Despite growing attention to health equity and aging, there remains limited conceptual clarity on what equitable aging entails in the context of migration. This concept analysis seeks to address this gap by exploring the defining attributes, antecedents, and consequences of equitable aging among migrants, providing a clearer foundation for research, policy development, and culturally responsive nursing practice. A careful concept analysis is needed to clarify equitable aging and inform targeted interventions and policy reforms that ensure dignified aging for all migrants. This analysis aims to: (a) conceptually define equitable aging among migrants and (b) develop a preliminary conceptual model to guide academic research and transcultural nursing care.

## Method

This study is informed by Walker and Avant (2019) concept analysis framework, a structured eight-phase methodology designed to systematically define and clarify key concepts. The analysis began by selecting the central concept (Step 1) and articulating the purpose of the analysis to develop a clear, practice-oriented definition that supports nursing theory and culturally responsive care (Step 2). Next, we conducted a targeted review of the literature to explore all known uses and meanings of the concept across disciplines and contexts (Step 3). Through this review, we identified key defining attributes that consistently characterize equitable aging (Step 4). To illustrate these attributes, we developed a model case that embodies all core features, as well as borderline and contrary cases to delineate conceptual boundaries (Step 5). We then analyzed the antecedents (preconditions) and consequences (outcomes) associated with equitable aging (Step 6). Finally, we identified empirical referents including existing tools and indicators used to observe and measure elements of the concept in practice (Step 7) and discussed their relevance to nursing care and policy development (Step 8). This structured process supports the development of a conceptual model that reflects the lived realities of aging migrant populations and enhances nursing practice. To enhance methodological rigor, we followed established literature review procedures, including the development of a comprehensive search strategy, systematic screening and data extraction, and thematic synthesis, while adhering to the PRISMA-ScR reporting guidelines (Page et al., 2021).

### Identifying the Concept, Relevant Concepts, and Search Strategy

The literature uses various terms to describe similar populations; this analysis adopts the term *migrant* as an inclusive label for immigrants, refugees, and undocumented individuals, consistent with the IOM definition (International Organization for Migration [IOM], 2019) of those relocating voluntarily or forcibly, permanently or temporarily. Key related concepts include aging with dignity, resourceful aging, and justice in aging also inform this analysis. The following definitions clarify key intersecting concepts—aging with dignity, resourceful aging, and justice in aging—that underpin the analysis of equitable aging among migrant populations. Aging with dignity involves maintaining autonomy, respect, and quality of life in later years, encompassing physical, emotional, and social well-being (Kisvetrová et al., 2022). It requires access to care, respect for personal values, and societal engagement (Kisvetrová et al., 2022; Theis et al., 2024; WHO, 2024). For migrants, this is often hindered by language barriers, limited health care access, and financial insecurity (Saltus & Pithara, 2014). Resourceful aging describes how older migrants adapt by drawing on resilience, cultural heritage, and social support to maintain dignity (Al-Hamad et al., 2025). Access to relevant health education, strong networks, and adaptive care strengthens this capacity (Rodrigues et al., 2023), aligning with holistic nursing’s focus on integrated care (Jasemi et al., 2017). Justice in aging emphasizes equitable treatment and access to resources for older adults, especially those from marginalized groups (Bastia et al., 2022). For migrants, this entails removing discriminatory barriers and ensuring culturally responsive care (Justice in Aging, 2025; The National CLAS Standards, 2024). Achieving justice requires policy reform and addressing systemic inequities (Kisvetrová et al., 2022; see Table 1 for Key Concepts Definition).

**Table 1.** Key Concepts Definitions.

Concept	Definition
Aging with dignity	Aging with dignity refers to the ability of older adults to maintain autonomy, respect, and quality of life as they grow older (Kisvetrová et al., 2022). It encompasses physical, emotional, and social well-being, ensuring that individuals receive appropriate care, respect for their personal values, and opportunities for meaningful engagement in society (Kisvetrová et al., 2022; Theis et al., 2024). World Health Organization (WHO, 2024) highlights that aging with dignity requires access to healthcare, financial security, and supportive environments that enable older adults to participate fully in their communities. For migrants, aging with dignity is often challenged by systemic barriers such as language limitations, healthcare inaccessibility, and financial insecurity (Saltus & Pithara, 2014).
Resourceful aging	Resourceful aging refers to older migrants adapting to aging by utilizing resources, embracing change, and maintaining resilience (Al-Hamad et al., 2025). This aging process balances cultural heritage with integration, enabling them to navigate aging with dignity while preserving identity, traditions, problem-solving skills, resilience, and the capacity to leverage social and community support systems (Al-Hamad et al., 2025). Access to culturally relevant health education, strong social networks, and adaptive healthcare strategies enhances their ability to navigate these challenges effectively (Rodrigues et al., 2023). Additionally, the concept of resourceful aging aligns with holistic nursing, which emphasizes treating the whole person by recognizing the interplay between physical, mental, and social health (Jasemi et al., 2017).
Justice in aging	Justice in aging is a social and ethical framework that advocates for fair treatment, rights, and access to resources for older adults, particularly those from marginalized or vulnerable populations (Bastia et al., 2022). It addresses structural inequalities in healthcare, housing, income security, and legal protections that affect aging populations (Justice in Aging, 2025). For older migrants, justice in aging means eliminating discriminatory policies that limit access to essential services and ensuring that healthcare systems provide culturally and linguistically appropriate care. The National CLAS Standards emphasize the need for equity and justice in healthcare services, aiming to reduce disparities and promote inclusivity for diverse aging populations (The National CLAS Standards, 2024). Ensuring justice in aging requires policy reforms, increased awareness, and commitment to addressing systemic barriers that prevent older adults from aging with dignity and security (Kisvetrová et al., 2022).

A comprehensive database search was conducted with support from a research librarian, using tailored keywords and Boolean strategies. The search was reviewed by another librarian using the PRESS framework (McGowan et al., 2016), with details for search strategy in Supplemental Appendix I. Google Scholar and reference lists were also searched to capture gray literature. The search strategy retrieved 351 records from multiple databases, including Web of Science (*n* = 109), CINAHL (*n* = 91), Medline (*n* = 22), SCOPUS (*n* = 81), PsycINFO (*n* = 48), and Google Scholar (*n* = 20). To maintain a contemporary of the recent waves of migration, only records published from 2010 to 2025 were included, reflecting the evolving migration patterns, aging policies, and support structures developed over the past two decades. The screening process was streamlined using Covidence software, which facilitated efficient record management and reviewer collaboration. After removing 22 duplicates and incomplete records using EndNote, 349 records were screened by two independent reviewers (RR and AZ). Studies were included if published in English and focused on older migrants. Disagreements were resolved through discussion or by a third reviewer (AA).

Following the PRISMA-ScR guidelines (Page et al., 2021), we conducted a systematic data extraction process to ensure consistency and transparency. A data extraction form was developed and piloted to capture relevant information from each included source, including author, year, context, population focus, conceptual definitions, methodological approaches, and key findings related to equitable aging. Two reviewers independently extracted data, and discrepancies were resolved through discussion and consensus. The extracted data were analyzed thematically analyzed (Braun & Clarke, 2023) to identify recurring patterns and refine the conceptual boundaries of equitable aging among migrants. This approach ensured a transparent link between literature and the emergent conceptual structure.

## Results

**Figure 1.**
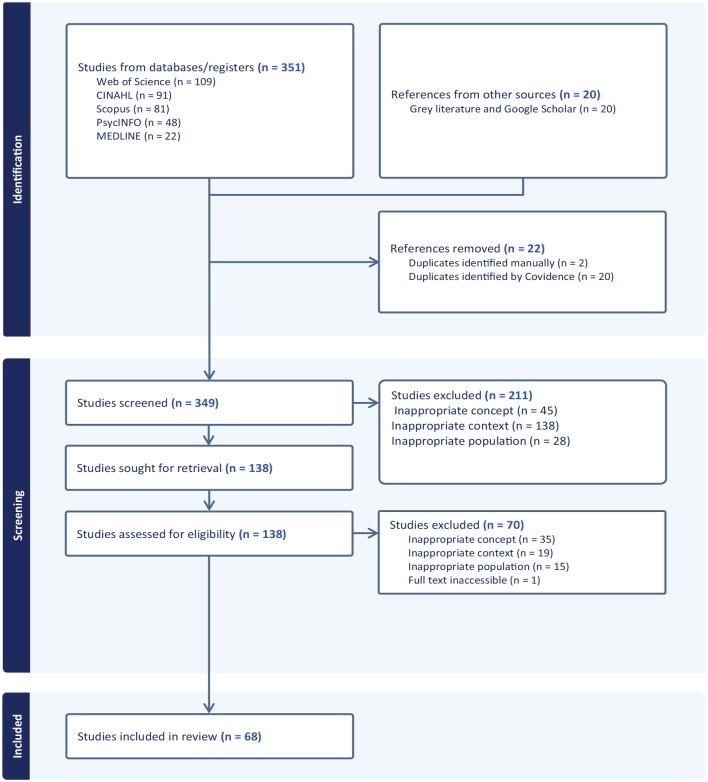
PRISMA Flow Diagram.

Of the 349 retrieved records, 211 were excluded, and 138 full-text articles were assessed. After further review, 70 were excluded, resulting in 68 studies included in the final analysis. No additional relevant sources were found through reference checks. The PRISMA-ScR diagram (Figure 1) details the screening process.

### Defining Attributes

According to Walker and Avant (2019), attributes represent the defining characteristics of a concept, serving as essential markers that differentiate it from related constructs. These attributes are crucial in determining the concept’s presence and ensuring conceptual clarity (Walker & Avant, 2019). Three fundamental attributes were identified in relation to equitable aging among migrants: (a) fair and just provision of health and social care services; (b) elimination of systemic barriers and disparities; and (c) inclusive and culturally responsive care.

#### Fair and Just Provision of Health and Social Care Services

Fair and just provision of health and social care services refer to the equitable delivery of health and social care services that actively address the cultural, linguistic, and systemic barriers faced by older migrants. Culturally responsive care is foundational to promoting accessibility and equity, particularly as cultural mismatches between migrants and mainstream health care systems often impede service access and utilization (Knipping et al., 2023; Kwan et al., 2016; Lai & Surood, 2013). Inclusive care must incorporate culturally relevant health education (Kwan et al., 2016), linguistically appropriate primary care (Sierra-Heredia et al., 2024), trauma-informed and family-centered supports (Jacobsen et al., 2023), and sensitive approaches to end-of-life care (Barragan-Carrillo et al., 2022). Additional domains such as oral health (Aarabi et al., 2018), mobility support (Newbold et al., 2023), and digital health literacy (Kaihlanen et al., 2022) must also reflect the cultural realities of migrant populations. The availability of culturally competent providers, language-trained professionals (Al Abed et al., 2014; Jacobsen et al., 2023; Nguyen & Reardon, 2013), and accessible health information in native languages (Meade et al., 2020) is essential to ensure that care is fair, just, and tailored to the diverse needs of aging migrant communities.

#### Elimination of Systemic Barriers and Disparities

Improving health care access for older migrants requires reducing waiting times, offering flexible hours, and ensuring geographic accessibility, particularly in underserved areas (Bolster-Foucault et al., 2024; Ding et al., 2025; Ghanbari-Jahromi et al., 2023; Kwan et al., 2016; Lai & Surood, 2013). Services must reflect the cultural and contextual needs of diverse groups to promote equity (Chowdhury et al., 2021). Migrants often face integration challenges, identity shifts, and exclusion, requiring community-based support (Georgeou et al., 2023; Siddiq et al., 2023). Economic insecurity and limited insurance further increase health risks, highlighting the need for affordable, inclusive services (Choi, 2011; Danso, 2016; Ma et al., 2021; Newbold et al., 2023; Ro et al., 2022).

Culturally trained interpreters and multilingual staff improve communication and care quality (Al Abed et al., 2014; Barragan-Carrillo et al., 2022; Jacobsen et al., 2023; Pot et al., 2020). Low health literacy limits care access and self-management, calling for multimodal education programs (Chen, 2010; Du & Xu, 2016; Ghanbari-Jahromi et al., 2023; Mandal & Pradhan, 2024). Accessible transport and home-based care further reduce barriers (Deckys & Springer, 2013; Lai & Surood, 2013).

#### Inclusive and Culturally Responsive Care

Inclusive, culturally responsive care requires language-accessible, culturally competent services that respect older migrants’ beliefs, values, and traditions (Balogun et al., 2025; Chui et al., 2020; Parker et al., 2024). Integrating traditional practices into Western care models can enhance accessibility and promote equity (Al Abed et al., 2014). Long-term care settings should adapt activities and food options to residents’ cultural preferences (Thao et al., 2025), while person-centered programs should align with linguistic and cultural needs (Lood et al., 2015). Health providers must also address mental health stigma, which may deter service use, by creating inclusive, non-judgmental environments (Giebel et al., 2015; Hunt et al., 2024; Siddiq et al., 2023). Sensitive topics like dementia, mental health, and end-of-life care require culturally tailored communication (Sayed et al., 2023). Trauma-informed care is essential for migrants with past distressing experiences (Georgeou et al., 2023), and employing providers with shared backgrounds can strengthen trust and engagement (Carlsson, 2023).

### Model and Additional Cases

To illustrate equitable aging among aging migrants, model, borderline, and contrary cases were developed (Table 2). A model case includes all defining attributes, while borderline and contrary cases reflect partial or incorrect examples of the concept (Walker & Avant, 2019).

**Table 2:** Model, Borderline and Contrary Cases.

Case	Definition	Developed case
Model case	Model case serves as an exemplary representation of the concept within real-world contexts, encompassing all the defining attributes identified in the analysis, as clarified by Walker and Avant (2019).	Khaled, a 68-year-old Syrian refugee, resettled after being displaced due to conflict. Upon arrival, he faced significant barriers to accessing healthcare, including language difficulties, unfamiliarity with the healthcare system, and financial constraints. However, a community health center specializing in refugee care provided him with tailored support, ensuring access to interpreters, multilingual health resources, and culturally sensitive consultations. Recognizing the psychological impact of displacement, the healthcare team incorporated mental health support and social services into his care plan. Additionally, government programs offered affordable healthcare, housing assistance, and financial aid, effectively removing the systemic obstacles that often hinder older refugees from receiving adequate care. Through these equitable healthcare measures, Khaled was able to navigate the healthcare system with confidence, maintain his well-being, and age with dignity in his new country.
Contrary case	A contrary case serves as an illustrative example that contrasts with the model case, providing a scenario that does not align with any of the defining attributes of the concept (Walker & Avant, 2019). This case offers an opposing perspective on equitable aging within the host country, demonstrating a situation where none of the defining attributes associated with the concept are present.	Zahra, a 70-year-old refugee woman from Afghanistan, arrived in a new country seeking safety and stability. However, she faced significant challenges in accessing healthcare and social support. Without adequate language assistance, she struggled to communicate with healthcare providers, making it difficult to receive proper medical care. The lack of culturally responsive services meant that her religious and personal health preferences were overlooked, leaving her feeling alienated and unsupported. Additionally, financial hardship and the absence of targeted assistance programs prevented her from securing stable housing or affording necessary medications. Systemic barriers such as bureaucratic complexity and limited outreach to refugee communities further marginalized her, resulting in delayed medical care, increased social isolation, and a decline in overall well-being. Zahra’s experience reflects the absence of fairness, inclusion, and systemic support, leaving her vulnerable and unable to age with dignity in her new environment.
Borderline case	A borderline case illustrates a situation in which only some attributes of the concept are present, but not all (Walker & Avant, 2019).	Danylo, a 69-year-old migrant from Ukraine, resettled in a new country after being displaced following the Russian invasion of Ukraine. While he was granted access to basic healthcare services, he faced challenges in navigating the healthcare system due to language barriers and unfamiliarity with available resources. A local community center provided limited interpretation services, which helped him communicate with doctors, but he still struggled with understanding complex medical procedures and health policies. Although he was eligible for government financial assistance, the bureaucratic process was overwhelming, causing delays in securing stable housing and consistent medical care. Culturally, he found some support within the Ukrainian expatriate community, which helped him maintain social connections and ease his transition. However, lacked culturally tailored healthcare approaches and services, and his specific needs as an older migrant were not fully addressed and prevented full accessibility to necessary services.

### Antecedents

Antecedents refer to the necessary conditions that must be in place for a concept to materialize, serving as the foundational elements that give rise to its defining attributes (Walker & Avant, 2019). For equitable aging to be achieved among older migrants, several key factors must be established beforehand. The following discussion illustrates the antecedents.

#### Availability of Accessible and Inclusive Services

Availability of Accessible and Inclusive Services is a foundational antecedent to equitable aging, as it reflects the structural capacity of systems to deliver fair, culturally responsive, and linguistically appropriate care to diverse aging populations. Accessible and inclusive services ensure fair access to aging support across diverse populations (Lood et al., 2015; Miyawaki et al., 2023). A wide range of affordable, culturally responsive services promotes choice and independence in care (Hurley et al., 2013). Addressing key aging needs such as primary care, cognitive and mental health, oral health, mobility, and cardiovascular care improves outcomes for older migrants (Aarabi et al., 2018; Hunt et al., 2024; Kwan et al., 2016; Newbold et al., 2023; Ogliari et al., 2020; Sadarangani, 2015; Shelley et al., 2011; Sierra-Heredia et al., 2024; Sung, 2014). Involving family caregivers boosts formal care acceptance, while interpreters, translators, and native-language resources support those with limited language skills (G. Kim et al., 2011; Sierra-Heredia et al., 2024; Sung, 2014).

#### Supportive Aging Policies and Frameworks

Supportive aging policies and frameworks are essential antecedents to equitable aging, as they establish the legal, institutional, and cultural infrastructure that shapes older migrants’ access to care, social inclusion, and financial security. Supportive aging policies and frameworks encompass the existing structural and societal components of systems that allow for equitable aging in older migrants (Salma & Salami, 2020). Universal health care is fundamental for assuring access to services, programs, interventions, and resources for all aging migrants (Abufaraj et al., 2024; Duguet et al., 2016). Cultural norms rooted in filial piety and expectations of intergenerational support foster family involvement in care and strengthen informal social networks (Abufaraj et al., 2024; Carlsson, 2023; Duguet et al., 2016). Legal protections in the form of citizenship status and policies against discrimination can safeguard against the exclusion of aging migrants in service access (Aarabi et al., 2018; S. Lin, 2024). Social security via pensions and benefits provide financial assistance for services and can reduce the financial burden of those in lower income brackets (Aarabi et al., 2018).

#### Familiarity With the Host Country’s Health Care System

Understanding how to navigate the host country’s health care system is essential for older migrants to access care and resources effectively (Coumans & Wark, 2024). Filial piety can aid in sharing health information within families (Xiao et al., 2024). Information should be offered in multiple formats include oral, audio, visual and reinforced across at least two types to accommodate low literacy, with traditional media often preferred over digital tools (Goodall et al., 2014). Culturally tailored education on disease prevention, such as cardiovascular care for Chinese migrants, is also vital (Kwan et al., 2016). Migrants should be informed about health care navigation and their rights as care recipients (Jang et al., 2020). Navigation programs, such as those for Hispanic patients in end-of-life care, improve service use and treatment outcomes (Barragan-Carrillo et al., 2022). Early mental health screening supports new migrants in accessing care (Siddiq et al., 2023), and outreach in trusted community or religious settings enhances service awareness (Okyerefo & Fiaveh, 2017).

#### Culturally Inclusive Community Engagement Initiatives

Culturally inclusive community engagement initiatives are vital antecedents to equitable aging, as they facilitate social inclusion, foster belonging, and empower older migrants to actively participate in their communities. Culturally inclusive community initiatives promote migrant participation and strengthen community ties (Runkle et al., 2012). Programs that link migrants with social support networks enhance inclusion and connectedness (Ding et al., 2025; van Hees et al., 2019). Mentorship from community leaders helps newcomers navigate systems and build trust (Cao et al., 2023; Koehn et al., 2022). Volunteering and community contributions foster belonging, while roles like cultural guides enhance older migrants’ self-worth (Kadowaki et al., 2023; Maleku et al., 2022). Incorporating skills like cooking or gardening, along with intergenerational programs, promotes engagement and reduces loneliness (Georgeou et al., 2023).

### Consequences

Consequences refer to the outcomes or implications that arise once a concept is fully realized, often leading to further exploration, policy considerations, or areas for future research (Walker & Avant, 2019). The subsequent discussion examines the potential effects and broader impact of equitable aging among migrants including.

#### Reduce Health and Social Disparities

Promoting equitable aging helps bridge long-standing health and social gaps for older migrants, fostering a more inclusive and just society. Equitable aging among older migrants significantly reduces health and social disparities, leading to greater inclusion and lower disparities between the older migrants in the host countries (Khoo, 2012; Lahaie et al., 2013; Q. Lin et al., 2022; Shelley et al., 2011). Enhanced access to health and social care not only improves quality of life but also promotes greater social integration and reduces health disparities (Chen, 2010; Duguet et al., 2016). Chowdhury et al. (2021) highlight the need for health care system adaptation to address language and cultural barriers, effectively reducing health and social care inequities for older migrants. Facilitating social networks and addressing structural inequities are crucial, leading to improved health outcomes and reduced social isolation (Johnson et al., 2021). Policies supporting cultural well-being and belonging among aging migrants enhance social participation while preserving cultural identity and facilitating integration (Georgeou et al., 2023).

#### Promote Health Equity and Service Utilization

Equitable aging facilitates greater health equity and empowers older migrants to access and utilize health and social services that meet their unique cultural and linguistic needs. Improving health equity and service access for older migrants reduces family burden and supports culturally influenced care decisions (Chen, 2010; Duguet et al., 2016; García-Ramírez et al., 2014). Better access leads to earlier diagnoses, timely interventions, and greater engagement in health programs (Duguet et al., 2016), while also fostering trust, autonomy, and dignity (Kisvetrová et al., 2022; Saltus & Pithara, 2014; Theis et al., 2024). Culturally responsive, affordable care with language support and respect for traditions improves quality of life and reduces disparities (Chen, 2010; García-Ramírez et al., 2014). Minority-specific services and providers enhance care acceptance (Carlsson, 2023). Removing service barriers and administrative burdens (Lai & Surood, 2013) and enacting inclusive health legislation (Floyd & Sakellariou, 2017) further promote health literacy and empower decision-making.

#### Foster Social Inclusion and Cultural Integration

Equitable aging contributes to fostering social inclusion and cultural integration, which are essential for enhancing the sense of belonging, reducing isolation, and promoting community engagement among older migrants. Effective social inclusion and cultural integration reduce isolation and improve community participation among older migrants (Johnson et al., 2021; Qi et al., 2022). Strengthening social networks and addressing structural inequities improve health outcomes and economic well-being (Johnson et al., 2021). Respecting cultural and religious traditions while expanding mental health access can reduce fear and isolation, especially among Middle Eastern/Arab American elders (Sayed et al., 2023). Combating racism, stereotypes, and language barriers enhances health care use and overall well-being (Al Abed et al., 2014). Social connectedness also boosts resilience, improving mental health and life satisfaction (Qi et al., 2022).

#### Improve Health and Enhance Quality of Life

Equitable aging plays a critical role in improving overall health and enhancing quality of life by ensuring that care is accessible, inclusive, and aligned with the cultural and linguistic needs of older migrants. Improved quality of life is a key outcome of equitable aging, supported by accessible, culturally responsive services (Vang, 2023; Vitman-Schorr & Khalaila, 2022; Zubritsky et al., 2013). Inclusive care reduces disparities, increases service use, and empowers older migrants through information and family support (Duguet et al., 2016). Access to timely care, insurance, and rural services further enhances well-being (Ghanbari-Jahromi et al., 2023). Respecting cultural preferences and eliminating structural racism in care facilities are also essential (Thao et al., 2025). Linguistically accessible services enable informed decision-making and promote autonomy, dignity, and personalized care (Kisvetrová et al., 2022; Nguyen & Reardon, 2013; Saltus & Pithara, 2014; Theis et al., 2024).

### Empirical Referents

The final step in concept analysis involves identifying empirical referents—observable indicators or measurement tools that reflect the presence and application of a concept in real-world settings (Walker & Avant, 2019). While no singular, validated instrument directly measures equitable aging among older migrants, several tools assess relevant dimensions that contribute to or constrain equity in aging. Structural determinants of health, such as access to services, language proficiency, and social support, are central to understanding inequities among migrant populations. The Commission of Social Determinants of Health Model (2008) provides a foundational framework for examining the societal and policy-level structures that shape health equity. Nguyen and Reardon (2013), for example, apply this model to highlight how language barriers contribute to systemic health disadvantages in aging migrant communities.

Psychological well-being, a key indicator of equitable aging, is assessed using validated tools such as the Kessler Psychological Distress Scale (K10), which evaluates nonspecific psychological distress (Furukawa et al., 2003), and the Geriatric Anxiety Inventory, which measures anxiety symptoms among older adults (Byrne & Pachana, 2011). Broader health-related quality of life is captured using the SF-12v2 Health Survey (Maruish, 2011), while the Connor-Davidson Resilience Scale (CD-RISC) evaluates psychological resilience, a protective factor in adapting to aging-related challenges (Connor & Davidson, 2003). In addition, the Composite International Diagnostic Interview–Short Form (CIDI-SF) screens for depressive symptoms and has been used in cross-cultural geriatric mental health studies (Gigantesco & Morosini, 2008). Social inclusion and connectedness, which are critical to equitable aging, are often measured by the Social Provisions Scale, capturing dimensions such as guidance, reassurance of worth, and social integration (Russell & Cutrona, 1984), and the De Jong Gierveld Loneliness Scale, which differentiates between emotional and social loneliness (De Jong Gierveld & Van Tilburg, 2010).

To contextualize these measures within diverse aging trajectories, broader frameworks also serve as empirical referents. The quality-of-life domains in long-term care, as outlined by Kane (2001, 2003), and applied by Zubritsky et al. (2013), provide measurable indicators of personal autonomy, dignity, and well-being within institutional settings. Khoo’s (2012) “Third Age” model conceptualizes aging as a phase of active contribution and personal growth, while Carlsson’s (2023) super-diversity framework underscores how multiple identity markers—such as ethnicity, gender, and migration history—intersect to shape older migrants’ access to equitable aging opportunities. Together, these empirical referents offer multidimensional tools and frameworks that, while not comprehensive on their own, collectively provide insight into how equitable aging may be identified, assessed, and promoted among older migrant populations.

### Conceptual Definition

Equitable aging among migrants refers to *the fair and inclusive process by which older migrants access culturally responsive care and supportive environments that uphold their health, dignity, and social inclusion.* This definition recognizes the diverse cultural, linguistic, and socioeconomic backgrounds of aging migrants achieved through supportive policies, community engagement, and culturally responsive services. Equitable aging promotes health equity, service utilization, and cultural integration, enabling aging migrants to experience later life with dignity, fostering their well-being and quality of life within host communities.

### Conceptual Model Development: Equitable Aging and Transcultural Nursing Care

**Figure 2.**
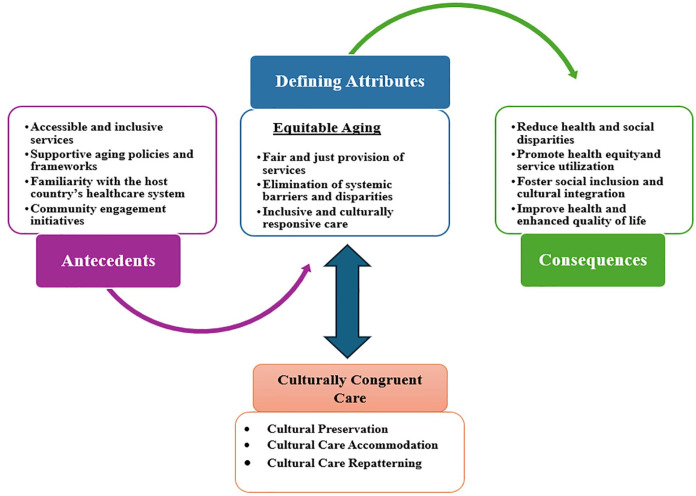
A Conceptual Model on Equitable Aging and Transcultural Nursing Care.

This section introduces a conceptual model linking equitable aging among older migrants with transcultural nursing. It explores how migrants navigate care and community life in host countries, highlighting conditions that support well-being, resilience, and quality of life. These themes align with transcultural nursing’s focus on inclusive, culturally congruent, community-based care (Im & Lee, 2018; Leininger, 2002; Tosun et al., 2021). The model (Figure 2) was developed collaboratively by four researchers (AA, RR, AZ, and YMY), with expert input enhancing its depth. While conceptually strong, it requires future quantitative validation. Rooted in Leininger’s theory that emphasizes cultural preservation, accommodation, and repatterning, the model promotes respectful, patient-centered care for aging migrants (Balogun et al., 2025; Leininger, 2002). See Table 3 for core principles of transcultural care.

**Table 3.** Core Principles of Transcultural Nursing Practice.

Principle	Discussion
Cultural preservation	Ensures that healthcare services respect and maintain older migrants’ cultural identities and traditions; many older migrants find comfort and stability in maintaining aspects of their cultural heritage, particularly in healthcare settings (Williams-Forson, 2018). Cultural preservation allows health and social care providers to support practices such as traditional healing methods, dietary customs, and religious observances, provided they do not interfere with medical safety (De Clercq, 2021). This is particularly important for older migrants who may feel alienated in a new cultural environment. For example, ensuring access to culturally appropriate foods in hospitals or long-term care facilities can enhance dietary compliance and nutritional well-being among aging migrant populations (Williams-Forson, 2018). Similarly, allowing space for prayer and religious rituals within health and social care settings can contribute to emotional and psychological well-being (Arrey et al., 2016; Okyerefo & Fiaveh, 2017; Ransford et al., 2010). By preserving these cultural elements, health and social care providers can foster a sense of belonging and security among older migrants, ultimately supporting their overall health and quality of life (De Clercq, 2021).
Cultural accommodation	Focuses on adapting health and social care practices to align with a patient’s cultural beliefs while maintaining medical effectiveness (Stanciu & Vauclair, 2018). Many older migrants face challenges in accessing healthcare due to language barriers (Pot et al., 2020), unfamiliar healthcare structures and differences in health-seeking behaviors (Kobayashi & Khan, 2023). Cultural accommodation strategies may include providing professional interpreters, offering multilingual health materials, and incorporating family members into healthcare decision-making (Piacentini et al., 2019). Additionally, flexible care plans that consider cultural practices, such as adjusting medication schedules around religious fasting periods or incorporating traditional healing approaches alongside conventional medical treatments can help bridge cultural gaps in healthcare (Ballout, 2024). This approach ensures that health and social care services remain accessible and responsive, thereby fostering trust and engagement among aging migrant populations (Pot et al., 2020).
Cultural repatterning	Involves modifying cultural practices that may negatively affect health outcomes while respecting the individual’s values and traditions (Pacquiao, 2008; Plane & Jurjevich, 2009). This principle is especially relevant in chronic disease management and preventive healthcare among older migrants (Sobon Sensor, 2019). For example, a healthcare provider may work with an older migrant patient to adjust high-sodium dietary preferences that contribute to hypertension while maintaining culturally familiar flavors and ingredients. Similarly, healthcare professionals may educate older migrants on the risks of certain traditional remedies while helping them find safer alternatives that align with their cultural health beliefs (Sobon Sensor, 2019). This approach ensures that older migrants receive medically sound care and medical advice without feeling that their cultural identity is being disregarded or undermined (Leininger, 2002).

## Discussion

The landscape of care emphasizes how older migrants navigate formal and informal networks, including culturally specific services (Khoo, 2012), community-based support (Peckham et al., 2018), and transnational health care practices (Metersky et al., 2024) all of which influence their integration into local care systems (S. Lin & Fang, 2023). However, aging migrants often face additional challenges such as mobility limitations (Garcia & Reyes, 2017), past experiences of discrimination (Kisvetrová et al., 2022), and evolving norms of filial piety (J. H. Kim & Silverstein, 2021), which affect their ability to access care. To promote equitable aging, policies should prioritize long-term trust-building with older migrant communities and adopt care landscape mapping to design culturally appropriate, linguistically accessible, and inclusive aged-care services (Hossen et al., 2023).

By shifting the focus from ethnic identity to broader migration-related variables, this approach provides a more nuanced and actionable strategy for improving aged-care accessibility among diverse migrant populations (Rodrigues et al., 2023). Despite the variety of methods to assess specific indicators and circumstances of aging among migrants, there are limited measures to delineate the unique concept of equity in aging migrants (AARP International, 2022; Balogun et al., 2025). The vulnerable positionality of aging migrants in the current socioeconomic and political landscape (Bastia et al., 2022) necessitates further research on appropriate evaluation of equity in the aging process.

The concept of equitable aging among older migrants emphasizes the need for a multidimensional approach that integrates social, cultural, and health care determinants to ensure fair and inclusive aging experiences (WHO, 2024). The preliminary conceptual model was informed by the principles of transcultural nursing, particularly cultural preservation, accommodation, and repatterning (Leininger, 2002). These principles highlight the necessity of culturally congruent care that respects older migrants’ identities while addressing systemic barriers that limit access to equitable care (Piacentini et al., 2019).

Our analysis revealed that equitable aging is shaped by several interrelated factors, including health care accessibility (Kobayashi & Khan, 2023), social support networks (Hossen et al., 2023), and culturally responsive policies (Sobon Sensor, 2019). Older migrants often face language barriers (Pot et al., 2020), unfamiliarity with health care systems (Rodrigues et al., 2023), and financial constraints (Szabó & Goodin, 2024) all of which affect their ability to receive adequate care. Addressing these challenges requires a transcultural nursing approach that prioritizes culturally and linguistically appropriate care (Piacentini et al., 2019), community engagement (Hossen et al., 2023), and person-centered health and social care delivery (Vakil et al., 2023). In addition, the cultural aspects of aging must be considered when implementing equitable health and social care strategies for older migrants (Stanciu & Vauclair, 2018).

The role of family, religion, and traditional healing practices in shaping health behaviors is critical (Ballout, 2024), and health and social care providers must balance cultural respect with medical best practices (Tosun et al., 2021). The preliminary conceptual model highlights the importance of cultural accommodation (Stanciu & Vauclair, 2018), cultural preservation (De Clercq, 2021) and cultural repatterning (Pacquiao, 2008; Plane & Jurjevich, 2009) to fit the beliefs and values of aging migrants while ensuring optimal health outcomes. By integrating transcultural nursing principles with the concept of equitable aging, the model offers a promising and comprehensive framework for addressing health disparities among aging migrants.

### Implications

The findings of this analysis have broad implications for policy, practice, research, and community action. Policymakers must develop inclusive aging policies that address the specific needs of older migrants, including access to health care, social services, and culturally tailored programs (Georgeou et al., 2023; Hossen et al., 2023; Kobayashi & Khan, 2023). Policies should also reduce systemic barriers such as language, finances, and bureaucratic complexity (Pot et al., 2020; Szabó & Goodin, 2024; Theis et al., 2024) and promote social inclusion (S. Lin, 2024).

In practice, transcultural care providers must deliver culturally congruent services through interpreter use, adapted materials, and respect for traditional practices (Ballout, 2024; Balogun et al., 2025; Im & Lee, 2018; Tosun et al., 2021). Interprofessional collaboration is vital for holistic care (Vakil et al., 2023).

The findings hold significant implications for transcultural nursing practice. Nurses play a pivotal role in promoting equitable aging by advocating for culturally responsive care that addresses the unique needs of aging migrant populations. Integrating the identified attributes into nursing assessment, care planning, and health promotion enables nurses to foster inclusion, reduce disparities, and support patient autonomy. Culturally competent nursing interventions, including the use of native-language resources, collaborative care models, and community engagement strategies, can enhance trust and service utilization among older migrants (Bastia et al., 2022; Rodrigues et al., 2023; Szabó & Goodin, 2024). In addition, nurses are well positioned to inform policy development and organizational change by highlighting systemic barriers that hinder equitable aging, thereby advancing the profession’s commitment to social justice and culturally safe care. Future research should explore lived migrant experiences, assess current interventions, and expand beyond English-language sources (Bastia et al., 2022; Rodrigues et al., 2023; Szabó & Goodin, 2024). Community organizations play a key role by providing culturally relevant support and bridging gaps between migrants and health systems (Al-Hamad et al., 2025; Baldassar & Wilding, 2020; Hossen et al., 2023; Qi et al., 2022).

### Limitations

This analysis views equitable aging among migrants as a complex, multidimensional concept shaped by socioeconomic, cultural, and policy factors. It focused on conceptual understanding rather than assessing literature quality. Due to its interdisciplinary nature, some aspects may not be fully captured, highlighting the need for further contextual research. The review was limited to English-language sources, potentially missing diverse regional perspectives. A key limitation of this concept analysis is the predominantly health care–focused literature base, which may have excluded relevant insights from disciplines such as sociology, law, and public policy that are integral to understanding equity in aging. In addition, while the proposed model offers a useful framework, it requires future validation and quantitative testing to confirm its relevance to transcultural nursing.

## Conclusion

This concept analysis used Walker and Avant’s (2019) method to examine equitable aging among older migrants, identifying key attributes, antecedents, and consequences. Equitable aging involves fair access to services, removal of systemic barriers, and culturally responsive care. Essential antecedents include accessible services, supportive policies, system familiarity, and community engagement. When present, these support well-being, social integration, and health care access. The findings offer a conceptual model to guide transcultural nursing and highlight gaps in policy and practice related to inclusivity and cultural competence. Further research should explore system adaptations across diverse sociocultural contexts to advance equitable aging.

## Supplemental Material

sj-docx-1-tcn-10.1177_10436596251359129 – Supplemental material for Equitable Aging Among Migrants: A Concept Analysis and Model Development for Transcultural Nursing Care Supplemental material, sj-docx-1-tcn-10.1177_10436596251359129 for Equitable Aging Among Migrants: A Concept Analysis and Model Development for Transcultural Nursing Care by Areej Al-Hamad, Yasin M. Yasin, Sepali Guruge, Kateryna Metersky, Lu Wang, Cristina Catallo, Hasina Amanzai, Zhixi Cecilia Zhuang, Rezwana Rahman and Andy Zhang in Journal of Transcultural Nursing

## References

[bibr1-10436596251359129] Aarabi G. Reissmann D. R. Seedorf U. Becher H. Heydecke G. Kofahl C. (2018). Oral health and access to dental care: A comparison of elderly migrants and non-migrants in Germany. Ethnicity & Health, 23(7), 703–717. 10.1080/13557858.2017.1294658 28277023

[bibr2-10436596251359129] Abufaraj M. Alhalaseh L. Al-sabbagh M. Q. Eyadat Z. Khatib W. A. Samara O. A. Moonesar I. A. Smith L. Al qutob R. (2024). The current status of health care indices and functional independence among older adults: Data from HelpAge international-Jordan study. Aging Clinical & Experimental Research, 36(1), 1–8. 10.1007/s40520-024-02738-2 38811496 PMC11136843

[bibr3-10436596251359129] Al Abed N. A. Davidson P. M. Hickman L. D . (2014). Healthcare needs of older Arab migrants: A systematic review. Journal of Clinical Nursing, 23(13), 1770–1784. 10.1111/jocn.12476 24329783

[bibr4-10436596251359129] Al-Hamad A. Yasin Y. Rahman R. Hayrabedian V. Metersky K. (2025). Resourceful aging among migrants and refugees: A concept analysis and model development for holistic nursing care. Journal of Holistic Nursing. Advance online publication. 10.1177/08980101251323012 PMC13149993 40095467

[bibr5-10436596251359129] American Association of Retired Persons (AARP) International. (2022). Achieving equitable healthy aging in low- and middle-income countries [The Aging Readiness & Competitiveness Report, Issue]. https://www.aarpinternational.org/file%20library/arc/aarp_economistimpact_arc4.0_report_final.pdf

[bibr6-10436596251359129] Arrey A. E. Bilsen J. Lacor P. Deschepper R. (2016). Spirituality/religiosity: A cultural and psychological resource among Sub-Saharan African migrant women with HIV/AIDS in Belgium. PLOS ONE, 11(7), e0159488. 10.1371/journal.pone.0159488 PMC4957758 27447487

[bibr7-10436596251359129] Ayón C. Ramos Santiago J. López Torres A. S. (2020). Latinx undocumented older adults, health needs and access to healthcare. Journal of Immigrant and Minority Health, 22, 996–1009. 10.1007/s10903-019-00966-7 31898077

[bibr8-10436596251359129] Baldassar L. Wilding R. (2020). Migration, aging, and digital kinning: The role of distant care support networks in experiences of aging well. The Gerontologist, 60(2), 313–321. 10.1093/geront/gnz156 31812983

[bibr9-10436596251359129] Ballout S. (2024). People of Arab Heritage. In Fenkl E. A. Purnell L. D. (Eds.), Handbook for culturally competent care (pp. 97–137). Springer. 10.1007/978-3-031-70492-5_8

[bibr10-10436596251359129] Balogun A. K. Atta J. A. Oyetubo O. M. Ibiam V. A. Bakare-Adesokan K. A. Ojo T. O. (2025). Developing culturally competent models for inclusive social work and healthcare interventions. International Journal of Science and Research Archive, 14(1), 1396–1406. 10.30574/ijsra.2025.14.1.0226

[bibr11-10436596251359129] Barragan-Carrillo R. Pabon C. M. Chavarri-Guerra Y. Soto-Perez-De-Celis E. Duma N. (2022). End-of-life care and advanced directives in Hispanic/Latinx patients: Challenges and solutions for the practicing oncologist. Oncologist, 27(12), 1074–1080. 10.1093/oncolo/oyac211 36288534 PMC10259759

[bibr12-10436596251359129] Bastia T. Lulle A. King R. (2022). Migration and development: The overlooked roles of older people and ageing. Progress in Human Geography, 46(4), 1009–1027. 10.1177/03091325221090535

[bibr13-10436596251359129] Bengtson V. (2018). Global aging and challenges to families. Routledge. 10.4324/9781351328166

[bibr14-10436596251359129] Bolster-Foucault C. Vedel I. Busa G. Hacker G. Sourial N. Quesnel-Vallée A. (2024). Social inequity in ageing in place among older adults in organization for economic cooperation and development countries: A mixed studies systematic review. Age & Ageing, 53(8), 1–17. 10.1093/ageing/afae166 PMC11321251 39137063

[bibr15-10436596251359129] Braun V. Clarke V. (2023). Thematic analysis. In Maggino F. (Ed.), Encyclopedia of quality of life and well-being research (pp. 7187–7193). Springer. 10.1007/978-3-031-17299-1_3470

[bibr16-10436596251359129] Byrne G. J. Pachana N. A. (2011). Development and validation of a short form of the Geriatric Anxiety Inventory–the GAI-SF. International Psychogeriatrics, 23(1), 125–131. 10.1017/S1041610210001237 20561386

[bibr17-10436596251359129] Cao Q. Krok-Schoen J. L. Guo M. Dong X. (2023). Trust in physicians, health insurance, and health care utilization among Chinese older immigrants. Ethnicity & Health, 28(1), 78–95. 10.1080/13557858.2022.2027881 35040724

[bibr18-10436596251359129] Carlsson H. (2023). Migrants’ pathways to aged care: The role of local relationships of care in facilitating access for super-diverse older populations. Ageing & Society, 43(7), 1502–1529. 10.1017/S0144686X21001240

[bibr19-10436596251359129] Chen M.-R. (2010). Access to health care and level of health-related quality of life of elderly Chinese immigrants in the Pacific Northwest [Doctoral dissertation, University of Washington]. ProQuest Dissertations & Theses Global. https://login.proxy.hil.unb.ca/login?url=https://search.ebscohost.com/login.aspx?direct=true&db=c8h&AN=109855029&site=ehost-live&scope=site

[bibr20-10436596251359129] Choi S. (2011). Longitudinal changes in access to health care by immigrant status among older adults: The importance of health insurance as a mediator. Gerontologist, 51(2), 156–169. 10.1093/geront/gnq064 20693237

[bibr21-10436596251359129] Chowdhury N. Naeem I. Ferdous M. Chowdhury M. Goopy S. Rumana N. Turin T. C. (2021). Unmet healthcare needs among migrant populations in Canada: Exploring the research landscape through a systematic integrative review. Journal of Immigrant & Minority Health, 23(2), 353–372. 10.1007/s10903-020-01086-3 32979131

[bibr22-10436596251359129] Chui C. H.-k. Arat G. Chan K. Wong P. W. C. (2020). Growing old as a member of an ethnic minority in Hong Kong: Implications for an inclusive long-term care policy framework. Journal of Applied Gerontology, 39(5), 463–471. 10.1177/0733464819873504 31496336

[bibr23-10436596251359129] The Commission of Social Determinants of Health Model. (2008). Closing the gap in a generation: Health equity through action on the social determinants of health: Commission on Social Determinants of Health final report. World Health Organization. https://www.who.int/publications/i/item/WHO-IER-CSDH-08.1

[bibr24-10436596251359129] Connor K. M. Davidson J. R. (2003). Connor–Davidson resilience scale. APA PsycTests. 10.1037/t06346-000 12964174

[bibr25-10436596251359129] Coumans J. V. F. Wark S. (2024). A scoping review on the barriers to and facilitators of health services utilization related to refugee settlement in regional or rural areas of the host country. BMC Public Health, 24(1), 199–209. 10.1186/s12889-024-17694-9 38229057 PMC10792843

[bibr26-10436596251359129] Danso K. (2016). Nativity and health disparities: Predictors of immigrant health. Social Work in Public Health, 31(3), 175–187. 10.1080/19371918.2015.1099494 26963922

[bibr27-10436596251359129] Deckys C. Springer P. (2013). The elderly Somali Bantu refugees’ adjustment to American healthcare. Online Journal of Cultural Competence in Nursing & Healthcare, 3(1), 3–15. 10.9730/ojccnh.org/v3n1a1

[bibr28-10436596251359129] De Clercq R . (2021). The importance of cultural preservation. In Allan Hillman T. Borland T. (Eds.), Dissident philosophers: Voices against the political current of the academy (pp. 107–121). Rowman & Littlefield.

[bibr29-10436596251359129] De Jong Gierveld J. Van Tilburg T . (2010). The De Jong Gierveld short scales for emotional and social loneliness: Tested on data from 7 countries in the UN generations and gender surveys. European Journal of Ageing, 7, 121–130. 10.1007/s10433-010-0144-6 20730083 PMC2921057

[bibr30-10436596251359129] De Voogd X. Oosterveld-Vlug M. Torensma M. Onwuteaka-Philipsen B. Willems D. Suurmond J . (2020). A dignified last phase of life for patients with a migration background: A qualitative study. Palliative Medicine, 34(10), 1385–1392. 10.1177/0269216320948708 32912088 PMC7543003

[bibr31-10436596251359129] Ding K. D. Ruksakulpiwat S. Wang Y. Voss J. G. (2025). The effects of residential segregation on cognition among US older adults: A systematic review based on the social determinants of health model. Aging & Mental Health, 29(1), 4–12. 10.1080/13607863.2024.2360016 38840518

[bibr32-10436596251359129] Du Y. Xu Q. (2016). Health disparities and delayed health care among older adults in California: A perspective from race, ethnicity, and immigration. Public Health Nursing, 33(5), 383–394. 10.1111/phn.12260 26990795

[bibr33-10436596251359129] Duguet A. M. Masmoudi T. Duchier J. Rwabihama J. P. Maatoug S. (2016). Access to care in France for elderly immigrants from North Africa: Influence of socio-cultural factors. European Journal of Health Law, 23(5), 470–480. 10.1163/15718093-12341431 29210247

[bibr34-10436596251359129] Floyd A. Sakellariou D. (2017). Healthcare access for refugee women with limited literacy: Layers of disadvantage. International Journal for Equity in Health, 16(1), 195–205. 10.1186/s12939-017-0694-8 29126420 PMC5681803

[bibr35-10436596251359129] Furukawa T. A. Kessler R. C. Slade T. Andrews G. (2003). The performance of the K6 and K10 screening scales for psychological distress in the Australian National Survey of Mental Health and Well-Being. Psychological Medicine, 33(2), 357–362. 10.1017/S0033291702006700 12622315

[bibr36-10436596251359129] Garcia M. A. Reyes A. M. (2017). Physical functioning and disability trajectories by age of migration among Mexican elders in the United States. The Journals of Gerontology: Series B, 73(7), 1292–1302. 10.1093/geronb/gbw167 PMC6146757 28052929

[bibr37-10436596251359129] García-Ramírez M. Balcázar F. de Freitas C. (2014). Community psychology contributions to the study of social inequalities, well-being and social justice. Psychosocial Intervention, 23(2), 79–81. 10.1016/j.psi.2014.07.009

[bibr38-10436596251359129] Georgeou N. Schismenos S. Wali N. Mackay K. Moraitakis E. (2023). A scoping review of aging experiences among culturally and linguistically diverse people in Australia: Toward better aging policy and cultural well-being for migrant and refugee adults. The Gerontologist, 63(1), 182–199. 10.1093/geront/gnab191 34969076 PMC9872767

[bibr39-10436596251359129] Ghanbari-Jahromi M. Bastani P. Jalali F. S. Delavari S. (2023). Factors affecting oral and dental services’ utilization among elderly: A scoping review. BMC Oral Health, 23(1), 597–609. 10.1186/s12903-023-03285-4 37635217 PMC10464329

[bibr40-10436596251359129] Giebel C. M. Zubair M. Jolley D. Bhui K. S. Purandare N. Worden A. Challis D. (2015). South Asian older adults with memory impairment: Improving assessment and access to dementia care. International Journal of Geriatric Psychiatry, 30(4), 345–356. 10.1002/gps.4242 25503751

[bibr41-10436596251359129] Gigantesco A. Morosini P. (2008). Development, reliability and factor analysis of a self-administered questionnaire which originates from the World Health Organization’s Composite International Diagnostic Interview–Short Form (CIDI-SF) for assessing mental disorders. Clinical Practice and Epidemiology in Mental Health, 4, 1–10. 10.1186/1745-0179-4-8 18402667 PMC2329624

[bibr42-10436596251359129] Glass A. P. Vander Plaats R. S. (2013). A conceptual model for aging better together intentionally. Journal of Aging Studies, 27(4), 428–442. 10.1016/j.jaging.2013.10.001 24300063

[bibr43-10436596251359129] Goodall K. T. Newman L. A. Ward P. R. (2014). Improving access to health information for older migrants by using grounded theory and social network analysis to understand their information behaviour and digital technology use. European Journal of Cancer Care, 23(6), 728–738. 10.1111/ecc.12241 25250535

[bibr44-10436596251359129] Henly S. J. (2016). Global migrations, ethical imperatives for care, and transcultural nursing research. Nursing Research, 65(5), 339. 10.1097/NNR.0000000000000181 27579501

[bibr45-10436596251359129] Hooten N. N. Pacheco N. L. Smith J. T. Evans M. K. (2022). The accelerated aging phenotype: The role of race and social determinants of health on aging. Ageing Research Reviews, 73, 101536. 10.1016/j.arr.2021.101536 34883202 PMC10862389

[bibr46-10436596251359129] Hossen M. Pauzi H. Salleh S. (2023). Enhancing elderly well-being through age-friendly community, social engagement and social support. American Journal of Science Education Research, 192, 1–10. 10.47991/2835-6764/AJSER-135

[bibr47-10436596251359129] Hunt M. G. Moro T. Stanley P. (2024). Unmet mental health care needs: Layered marginalities in older adult populations. Generations, 48(1), 1–11. https://www.jstor.org/stable/48794567

[bibr48-10436596251359129] Hurley C. Panagiotopoulos G. Tsianikas M. Newman L. Walker R. (2013). Access and acceptability of community-based services for older Greek migrants in Australia: User and provider perspectives. Health & Social Care in the Community, 21(2), 140–149. 10.1111/hsc.12000 23009742

[bibr49-10436596251359129] Im E.-O. Lee Y. (2018). Transcultural nursing: Current trends in theoretical work. Asian Nursing Research, 12(3), 157–165. 10.1016/j.anr.2018.08.006 30179700

[bibr50-10436596251359129] International Organization for Migration. (2019). Migrant. IOM Glossary on Migration. https://publications.iom.int/system/files/pdf/iml_34_glossary.pdf

[bibr51-10436596251359129] International Organization for Migration. (2024). Key migration terms. https://www.iom.int/key-migration-terms

[bibr52-10436596251359129] Jacobsen F. F. Glasdam S. Schopman L. M. Sodemann M. van den Muijsenbergh M. E. Ågotnes G. (2023). Migration and health: Exploring healthy ageing of immigrants in European societies. Primary Health Care Research & Development, 24, e10. 10.1017/S1463423623000014 PMC9971849 36733211

[bibr53-10436596251359129] Jang Y. Park N. S. Chiriboga D. A. Rhee M.-K. Yoon H. Kim M. T. (2020). Healthcare navigation self-sufficiency in older Korean immigrants. Journal of Applied Gerontology, 39(5), 457–462. 10.1177/0733464819842495 30983477 PMC7112185

[bibr54-10436596251359129] Jasemi M. Valizadeh L. Zamanzadeh V. Keogh B. (2017). A concept analysis of holistic care by hybrid model. Indian Journal of Palliative Care, 23(1), 71–80. 10.4103/0973-1075.197960 28216867 PMC5294442

[bibr55-10436596251359129] Johnson S. Bacsu J. McIntosh T. Jeffery B. Novik N. (2021). Competing challenges for immigrant seniors: Social isolation and the pandemic. Healthcare Management Forum, 34(5), 266–271. 10.1177/08404704211009233 33982605 PMC8127016

[bibr56-10436596251359129] Justice in Aging. (2025). Fighting senior poverty through the law. https://justiceinaging.org/

[bibr57-10436596251359129] Kadowaki L. Koehn S. D. Brotman S. Simard J. Ferrer I. Raymond É. Orzeck P. (2023). Learning from the lived experiences of aging immigrants: Extending the reach of photovoice using world cafe methods. Journal of Community Engagement and Scholarship, 16(1), 4. 10.54656/jces.v16i1.551

[bibr58-10436596251359129] Kaihlanen A.-M. Virtanen L. Buchert U. Safarov N. Valkonen P. Hietapakka L. Horhammer I. Kujala S. Kouvonen A. Heponiemi T. (2022). Towards digital health equity: A qualitative study of the challenges experienced by vulnerable groups in using digital health services in the COVID-19 era. BMC Health Services Research, 22(1), 188. 10.1186/s12913-022-07584-4 35151302 PMC8840681

[bibr59-10436596251359129] Kane R. A. (2001). Long-term care and a good quality of life: Bringing them closer together. The Gerontologist, 41(3), 293–304. 11405425 10.1093/geront/41.3.293

[bibr60-10436596251359129] Kane R. A. (2003). Definition, measurement, and correlates of quality of life in nursing homes: Toward a reasonable practice, research, and policy agenda. The Gerontologist, 43(2), 28–36. 10.1093/geront/43.suppl_2.28 12711722

[bibr61-10436596251359129] Kc S. Clarke K. Seppänen M. (2024). “If I count everything that is against me. It is my color. It is that I am a woman”: Exploring the lived experiences of racialized older migrant women in Finland. The British Journal of Social Work, 54(1), 22–39. 10.1093/bjsw/bcad178

[bibr62-10436596251359129] Khoo S.-E. (2012). Ethnic disparities in social and economic well-being of the immigrant aged in Australia. Journal of Population Research, 29, 119–140. 10.1007/s12546-012-9080-y

[bibr63-10436596251359129] Kim G. Worley C. B. Allen R. S. Vinson L. Crowther M. R. Parmelee P. Chiriboga D. A. (2011). Vulnerability of older Latino and Asian immigrants with limited English proficiency. Journal of the American Geriatrics Society, 59(7), 1246–1252. 10.1111/j.1532-5415.2011.03483.x 21718269

[bibr64-10436596251359129] Kim J. H. Silverstein M. (2021). Are filial piety and ethnic community engagement associated with psychological wellbeing among older Chinese American immigrants? A cultural resource perspective. Research on Aging, 43(2), 63–73. 10.1177/0164027520937477 32662345

[bibr65-10436596251359129] Kisvetrová H. Mandysová P. Tomanová J. Steven A. (2022). Dignity and attitudes to aging: A cross-sectional study of older adults. Nursing Ethics, 29(2), 413–424. 10.1177/09697330211057223 34875911 PMC8958642

[bibr66-10436596251359129] Knipping D. Garnett A. Jiang B. B. (2023). Access and use of services by caregivers of older adults: A scoping review of cultural and linguistic diversity. Journal of Applied Gerontology, 42(7), 1672–1686. 10.1177/07334648231158490 36866817 PMC10272625

[bibr67-10436596251359129] Kobayashi K. M. Khan M. M. (2023). Older migrants and access and usage of care. In Torres S. Hunter A. (Eds.), Handbook on migration and ageing (pp. 311–321). Edward Elgar. 10.4337/9781839106774.00038

[bibr68-10436596251359129] Koehn S. D. Donahue M. Feldman F. Drummond N. (2022). Fostering trust and sharing responsibility to increase access to dementia care for immigrant older adults. Ethnicity & Health, 27(1), 83–99. 10.1080/13557858.2019.1655529 31416342

[bibr69-10436596251359129] Kwan C. Lin Y. S. Homel P. Rojas M. Shetty V. Lichstein E. (2016). Barriers to care in elderly Chinese adults with heart disease (pp. 41–42). Wiley-Blackwell. 10.1111/jgs.14329 27562949

[bibr70-10436596251359129] Lahaie C. Earle A. Heymann J. (2013). An uneven burden: Social disparities in adult caregiving responsibilities, working conditions, and caregiver outcomes. Research on Aging, 35(3), 243–274. 10.1177/0164027512446028

[bibr71-10436596251359129] Lai D. W. L. Surood S. (2013). Effect of service barriers on health status of aging South Asian immigrants in Calgary, Canada. Health and Social Work, 38(1), 41–50. 10.1093/hsw/hls065 23539895

[bibr72-10436596251359129] Lebrun L. A. Shi L. (2011). Nativity status and access to care in Canada and the US: Factoring in the roles of race/ethnicity and socioeconomic status. Journal of Health Care for the Poor and Underserved, 22(3), 1075–1100. 10.1353/hpu.2011.0075 21841297

[bibr73-10436596251359129] Leininger M. (2002). Transcultural nursing and globalization of health care: Importance, focus, and historical aspects. Transcultural Nursing: Concepts, Theories, Research and Practice, 3, 3–43.

[bibr74-10436596251359129] Lin Q. Paykin S. Halpern D. Martinez-Cardoso A. Kolak M. (2022). Assessment of structural barriers and racial group disparities of covid-19 mortality with spatial analysis. JAMA Network Open, 5(3), e220984. 10.1001/jamanetworkopen.2022.0984 PMC8897755 35244703

[bibr75-10436596251359129] Lin S. (2024). Immigrant and racialized populations’ cumulative exposure to discrimination and associations with long-term conditions during covid-19: A nationwide large-scale study in Canada. Journal of Racial and Ethnic Health Disparities, 12, 2607–2622. 10.1007/s40615-024-02074-1 39017775 PMC12241279

[bibr76-10436596251359129] Lin S. Fang L. (2023). Chronic care for all? The intersecting roles of race and immigration in shaping multimorbidity, primary care coordination, and unmet health care needs among older Canadians. The Journals of Gerontology: Series B, 78(2), 302–318. 10.1093/geronb/gbac125 36044754

[bibr77-10436596251359129] Lood Q. Häggblom-Kronlöf G. Dahlin-Ivanoff S. (2015). Health promotion program design and efficacy in relation to ageing persons with culturally and linguistically diverse backgrounds: A systematic literature review and meta-analysis. BMC Health Services Research, 15, 560–570. 10.1186/s12913-015-1222-4 26674647 PMC4682220

[bibr78-10436596251359129] Ma K. P. K. Bacong A. M. Kwon S. C. Yi S. S. Doan L. N. (2021). The impact of structural inequities on older Asian Americans during COVID-19. Frontiers in Public Health, 9, 690014. 10.3389/fpubh.2021.690014 34490181 PMC8417937

[bibr79-10436596251359129] Maleku A. España M. Jarrott S. Karandikar S. Parekh R. (2022). We are aging too! Exploring the social impact of late-life migration among older immigrants in the United States. Journal of Immigrant & Refugee Studies, 20(3), 365–382. 10.1080/15562948.2021.1929643

[bibr80-10436596251359129] Mandal B. Pradhan K. C. (2024). A comparative study of health outcomes between elderly Migrant and non-migrant population in India: Exploring health disparities through propensity score matching. SSM—Population Health, 25, 101619. 10.1016/j.ssmph.2024.101619 38371497 PMC10869293

[bibr81-10436596251359129] Maruish M. E. (2011). User’s manual for the SF-36v2 Health Survey (3rd ed.). QualityMetric.

[bibr82-10436596251359129] McGowan J. Sampson M. Salzwedel D. M. Cogo E. Foerster V. Lefebvre C. (2016). PRESS peer review of electronic search strategies: 2015 guideline statement. Journal of Clinical Epidemiology, 75, 40–46. 10.1016/j.jclinepi.2016.01.021 27005575

[bibr83-10436596251359129] Meade C. D. Christy S. M. Gwede C. K. (2020). Improving communications with older cancer patients. In Extermann M. (Ed.), Geriatric oncology (pp. 991–1013). 10.1007/978-3-319-57415-8_21

[bibr84-10436596251359129] Metersky K. Guruge S. Wang L. Al-Hamad A. Yasin Y. M. Catallo C. Yang L. Salma J. Zhuang Z. C. Chahine M. Kirkwood M. Al-Anani A. (2024). Transnational healthcare practices among migrants: A concept analysis. Journal of Advanced Nursing, 81, 3647–3673. 10.1111/jan.16693 39722540 PMC12159394

[bibr85-10436596251359129] Miyawaki C. E. Garcia J. M. Nguyen K. N. Park V. T. Markides K. S. (2023). Multiple chronic conditions and disability among Vietnamese older adults: Results from the Vietnamese Aging and Care Survey (VACS). Journal of Racial and Ethnic Health Disparities, 11, 1800–1807. 10.1007/s40615-023-01652-z 37249829 PMC11006017

[bibr86-10436596251359129] The National CLAS Standards. (2024). National culturally and linguistically appropriate services standards. https://thinkculturalhealth.hhs.gov/clas/standards

[bibr87-10436596251359129] Newbold K. B. Valaitis R. Phillips S. Alvarez E. Neil-Sztramko S. Sihota D. Tandon M. Nadarajah A. Wang A. Moore C. Orr E. Ganann R. (2023). Enhancing physical and community mobility in older adults with health inequities using community co-design (EMBOLDEN): Results of an environmental scan. Canadian Geriatrics Journal, 26(1), 23–30. 10.5770/cgj.26.602 36865406 PMC9953504

[bibr88-10436596251359129] Nguyen D. Reardon L. J. (2013). The role of race and English proficiency on the health of older immigrants. Social Work in Health Care, 52(6), 599–617. 10.1080/00981389.2013.772554 23865974

[bibr89-10436596251359129] Ogliari G. Turner Z. Khalique J. Gordon A. L. Gladman J. R. F. Chadborn N. H. (2020). Ethnic disparity in access to the memory assessment service between South Asian and White British older adults in the United Kingdom: A cohort study. International Journal of Geriatric Psychiatry, 35(5), 507–515. 10.1002/gps.5263 31943347

[bibr90-10436596251359129] Okyerefo M. P. K. Fiaveh D. Y. (2017). Prayer and health-seeking beliefs in Ghana: Understanding the religious space of the urban forest. Health Sociology Review, 26(3), 308–320. 10.1080/14461242.2016.1257360

[bibr91-10436596251359129] Pacquiao D. F. (2008). Nursing care of vulnerable populations using a framework of cultural competence, social justice and human rights. Contemporary Nurse, 28(1), 189–197. 10.5172/conu.673.28.1-2.189 18844572

[bibr92-10436596251359129] Page M. J. McKenzie J. E. Bossuyt P. M. Boutron I. Hoffmann T. C. Mulrow C. D. Shamseer L. Tetzlaff J. M. Akl E. A. Brennan S. E. Chou R. Glanville J. Grimshaw J. M. Hróbjartsson A. Lalu M. M. Li T. Loder E. W. Mayo-Wilson E. McDonald S. . . . Moher D. (2021). The PRISMA 2020 statement: An updated guideline for reporting systematic reviews. Journal of Clinical Epidemiology, 134, 178–189. 10.1016/j.jclinepi.2021.03.001 33789819

[bibr93-10436596251359129] Parker E. Schut R. A. Boen C. (2024). The promise and limits of inclusive public policy: Federal safety net clinics and immigrant access to health care in the US. Social Forces, 103(3), 992–1017. 10.1093/sf/soae111 PMC11726819 39811565

[bibr94-10436596251359129] Peckham A. Rudoler D. Li J. M. D’souza S. (2018). Community-based reform efforts: The case of the aging at home strategy. Healthcare Policy, 14(1), 30–43. 10.12927/hcpol.2018.25550 30129433 PMC6147369

[bibr95-10436596251359129] Piacentini T. O’Donnell C. Phipps A. Jackson I. Stack N. (2019). Moving beyond the “language problem”: Developing an understanding of the intersections of health, language and immigration status in interpreter-mediated health encounters. Language and Intercultural Communication, 19(3), 256–271. 10.1080/14708477.2018.1486409

[bibr96-10436596251359129] Plane D. A. Jurjevich J. R. (2009). Ties that no longer bind? The patterns and repercussions of age-articulated migration. The Professional Geographer, 61(1), 4–20. 10.1080/00330120802577558

[bibr97-10436596251359129] Pot A. Keijzer M. De Bot K. (2020). The language barrier in migrant aging. International Journal of Bilingual Education and Bilingualism, 23(9), 1139–1157. 10.1080/13670050.2018.1435627

[bibr98-10436596251359129] Prosen M. Karnjuš I. Ličen S. (2021). Introduction to transcultural nursing. In Yava A. Tosun B. (Eds.), Transcultural nursing: Better & effective nursing education for improving transcultural nursing skills (Benefits) (pp. 9–18). Ankara Tıp Nobel Kitabevleri.

[bibr99-10436596251359129] Qi X. Zhang W. Wang K. Pei Y. Wu B. (2022). Social isolation and psychological well-being among older Chinese Americans: Does resilience mediate the association? International Journal of Geriatric Psychiatry, 37(8), 1–9. 10.1002/gps.5791 35866312

[bibr100-10436596251359129] Ransford H. E. Carrillo F. R. Rivera Y. (2010). Health care-seeking among Latino immigrants: Blocked access, use of traditional medicine, and the role of religion. Journal of Health Care for the Poor and Underserved, 21(3), 862–878. 10.1353/hpu.0.0348 20693732

[bibr101-10436596251359129] Ro A. Van Hook J. Walsemann K. M. (2022). Undocumented older Latino immigrants in the United States: Population projections and share of older undocumented Latinos by health insurance coverage and chronic health conditions. The Journals of Gerontology: Series B, 77(2), 389–395. 10.1093/geronb/gbab189 34644384

[bibr102-10436596251359129] Rodrigues C. E. Grandt C. L. Alwafa R. A. Badrasawi M. Aleksandrova K. (2023). Determinants and indicators of successful aging as a multidimensional outcome: A systematic review of longitudinal studies. Frontiers in Public Health, 11, 1258280. 10.3389/fpubh.2023.1258280 38074742 PMC10703300

[bibr103-10436596251359129] Runkle J. D. Brock-Martin A. Karmaus W. Svendsen E. R. (2012). Secondary surge capacity: A framework for understanding long-term access to primary care for medically vulnerable populations in disaster recovery. American Journal of Public Health, 102(12), 24–32. 10.2105/AJPH.2012.301027 PMC3519329 23078479

[bibr104-10436596251359129] Russell D. W. Cutrona C. E. (1984). Social Provisions Scale. Iowa State University.

[bibr105-10436596251359129] Sadarangani T. R. (2015). Policy implications of a literature review of cardiovascular disease in uninsured immigrant older adults. Journal of Gerontological Nursing, 41(6), 14–20. 10.3928/00989134-20150410-01 25912238

[bibr106-10436596251359129] Salma J. Salami B. (2020). “We are like any other people, but we don’t cry much because nobody listens”: The need to strengthen aging policies and service provision for minorities in Canada. Gerontologist, 60(2), 279–290. 10.1093/geront/gnz184 31944237

[bibr107-10436596251359129] Saltus R. Pithara C. (2014). A sense of dignity in later life: A qualitative study on the views of older women migrants from minoritized backgrounds. Quality in Ageing and Older Adults, 15(1), 21–33. 10.1108/QAOA-06-2013-0016

[bibr108-10436596251359129] Sayed L. Alanazi M. Ajrouch K. J. (2023). Self-reported cognitive aging and well-being among older middle Eastern/Arab American immigrants during the COVID-19 pandemic. International Journal of Environmental Research and Public Health, 20(11), 5918. 10.3390/ijerph20115918 37297521 PMC10252503

[bibr109-10436596251359129] Sen A. Nagaram S. (2024). Navigating the intersection of aging and disability. In Bennett G. Goodall E. (Eds.), The Palgrave encyclopedia of disability (pp. 1–16). Springer. 10.1007/978-3-031-40858-8_375-1

[bibr110-10436596251359129] Settersten R. A. Angel J. L. (2011). Handbook of sociology of aging. Springer.

[bibr111-10436596251359129] Shelley D. Russell S. Parikh N. S. Fahs M. Shelley D. Russell S. Parikh N. S. Fahs M. (2011). Ethnic disparities in self-reported oral health status and access to care among older adults in NYC. Journal of Urban Health, 88(4), 651–662. 10.1007/s11524-011-9555-8 21850607 PMC3157507

[bibr112-10436596251359129] Siddiq H. Elhaija A. Wells K. (2023). An integrative review of community-based mental health interventions among resettled refugees from Muslim-majority countries. Community Mental Health Journal, 59(1), 160–174. 10.1007/s10597-022-00994-y 35751790 PMC9244342

[bibr113-10436596251359129] Sierra-Heredia C. Tayyar E. Bozorgi Y. Thakore P. Hagos S. Carrillo R. Machado S. Peterson S. Goldenberg S. Wiedmeyer M.-l. Lavergne M. R. (2024). Growing inequities by immigration group among older adults: Population-based analysis of access to primary care and return to in-person visits during the COVID-19 pandemic in British Columbia, Canada. BMC Primary Care, 25(1), 1–12. 10.1186/s12875-024-02530-1 39243016 PMC11378608

[bibr114-10436596251359129] Sobon Sensor C . (2019). Health-related beliefs, practices, and experiences of migrant Dominicans in the Northeastern United States. Journal of Transcultural Nursing, 30(5), 492–500. 10.1177/1043659618801967 30284499

[bibr115-10436596251359129] Stanciu A. Vauclair C.-M. (2018). Stereotype accommodation: A socio-cognitive perspective on migrants’ cultural adaptation. Journal of Cross-Cultural Psychology, 49(7), 1027–1047. 10.1177/0022022118777300

[bibr116-10436596251359129] Sung J. (2014). What limits access to speech-language pathology services in the Asian elderly community? Perspectives on Gerontology, 19(3), 87–99. 10.1044/gero19.3.87

[bibr117-10436596251359129] Szabó Á. Goodin R. E . (2024). Comparing the health status of immigrant and New Zealand-born older adults in Aotearoa New Zealand: The role of socioeconomic position. Journal of Aging and Health. Advance online publication. 10.1177/08982643241276268 PMC12541109 39422625

[bibr118-10436596251359129] Tang S. Long C. Wang R. Liu Q. Feng D. Feng Z. (2020). Improving the utilization of essential public health services by Chinese elderly migrants: Strategies and policy implication. Journal of Global Health, 10(1), 1–11. 10.7189/jogh.10.010807 PMC7125420 32257170

[bibr119-10436596251359129] Thao M. S. Davila H. Shippee T. (2025). “I feel like a caged pig in here”: Language, race, and ethnic identity in a case study Hmong nursing home resident quality of life. Journal of Applied Gerontology, 44(2), 267–275. 10.1177/07334648241271896 39140747 PMC11758891

[bibr120-10436596251359129] Theis D. Wise B. Sprinkle B. Bainter T. (2024). Maintaining dignity and autonomy in the process of aging. The American Journal of Geriatric Psychiatry, 32(4), S16. 10.1016/j.jagp.2024.01.065

[bibr121-10436596251359129] Tosun B. Yava A. Dirgar E. Şahin E. B. Yılmaz E. B. Papp K. Tóthova V. Hellerova V. Prosen M. Licen S. (2021). Addressing the effects of transcultural nursing education on nursing students’ cultural competence: A systematic review. Nurse Education in Practice, 55, 103171. 10.1016/j.nepr.2021.103171 34388616

[bibr122-10436596251359129] Vakil K. Desse T. A. Manias E. Alzubaidi H. Rasmussen B. Holton S. Mc Namara K. P. (2023). Patient-centered care experiences of first-generation, South Asian migrants with chronic diseases living in high-income, Western countries: Systematic review. Patient Preference and Adherence, 17, 281–298. 10.2147/PPA.S391340 36756536 PMC9899934

[bibr123-10436596251359129] Vang S. (2023). Health-related quality of life in elderly Asian American and non-Hispanic White cancer survivors. Journal of Preventive Medicine & Public Health, 56(5), 440–448. 10.3961/jpmph.22.464 37735828 PMC10579642

[bibr124-10436596251359129] van Hees S. G. M. O’Fallon T. Hofker M. Dekker M. Polack S. Banks L. M. Spaan E. J. A. M . (2019). Leaving no one behind? Social inclusion of health insurance in low- and middle-income countries: A systematic review. International Journal for Equity in Health, 18(1), 134–153. 10.1186/s12939-019-1040-0 31462303 PMC6714392

[bibr125-10436596251359129] Vitman-Schorr A. Khalaila R. (2022). Utilization of ADCCs and quality of life among older adults: Ethno-regional disparities in Israel. BMC Geriatrics, 22(1), 18. 10.1186/s12877-021-02674-0 34979954 PMC8722010

[bibr126-10436596251359129] Walker O. Avant C. (2019). Strategies for theory construction in nursing (6th ed.). Pearson.

[bibr127-10436596251359129] Williams-Forson P. (2018). “I haven’t eaten if I don’t have my soup and fufu”: Cultural preservation through food and foodways among Ghanaian migrants in the United States. In Counihan C. Esterik P. V. (Eds.), Food and culture (pp. 205–220). Routledge. 10.2979/africatoday.61.1.69

[bibr128-10436596251359129] World Health Organization. (2024). Ageing and health. https://www.who.int/news-room/fact-sheets/detail/ageing-and-health

[bibr129-10436596251359129] Xiao C. H. Patrician P. A. Montgomery A. P. Wang Y. H. Jablonski R. Markaki A. (2024). Filial piety and older adult caregiving among Chinese and Chinese-American families in the United States: A concept analysis. BMC Nursing, 23(1), 1–12. 10.1186/s12912-024-01789-0 38347512 PMC10863110

[bibr130-10436596251359129] Zubritsky C. Abbott K. M. Hirschman K. B. Bowles K. H. Foust J. B. Naylor M. D. (2013). Health-related quality of life: Expanding a conceptual framework to include older adults who receive long-term services and support. The Gerontologist, 53(2), 205–210. 10.1093/geront/gns093 22859435 PMC3695648

