# Supplementary material for: Equitable Aging Among Migrants: A Concept Analysis and Model Development for Transcultural Nursing Care
Source: J Transcult Nurs. 2025 Aug 12;36(6):644–60. doi: 10.1177/10436596251359129 (PMC12521773; doi:10.1177/10436596251359129)
Supplement: sj-docx-1-tcn-10.1177_10436596251359129 – Supplemental material for Equitable Aging Among Migrants: A Concept Analysis and Model Development for Transcultural Nursing Care [file sj-docx-1-tcn-10.1177_10436596251359129.docx]

**Appendix I: Search Terms and Search Strategy**

**(English language (2010-2025), age above 60 years old)**

**Search Databases:**

Medline

CINAHL (Cumulative Index to Nursing and Allied Health Literature)

PsycINFO (Psychology and Social Sciences)

Scopus

Web of Science

ProQuest Social Science Collection.

**Keywords and Search Terms**

**Aging-related terms:**

"Aging" OR "ageing"

"Older adults" OR "elderly" OR "senior citizens"

"Geriatrics" OR "aging population" limit 60

**Migrant-related terms:**

"Migrant" OR "immigrant" OR "refugee" OR "displaced persons"

OR "Newcomers" OR "foreign-born"

**Equity-related terms:**

"Equity" OR "equitable" OR "fairness"

"Social justice" OR "health equity" OR "disparities"

"Access to care" OR "inclusive services"

**healthcare-related terms:**

"Healthcare access" OR "health services utilization"

"Well-being" OR "quality of life" OR "support systems"

"Culturally competent care" OR “ determinants of health”

("Aging" OR "older adults" OR "elderly") AND ("Migrant" OR "immigrant" OR "refugee") AND ("Equity" OR "social justice" OR "health disparities") AND ("Healthcare access" OR "social inclusion" OR "economic security")

("Health equity" OR "health disparities") AND ("Older adults" OR "senior citizens") AND ("Immigrants" OR "refugees") AND ("Culturally competent care" OR "access to healthcare")

Social participation" OR "social inclusion") AND ("Aging migrants" OR "foreign-born older adults") AND ("Community support" OR "policy interventions")

| Data base | Search strategy | Founded articles |
| --- | --- | --- |
| Medline through Ovid | (aging OR ageing OR "geriatrics" OR "aging population" OR "older adults" OR "elderly" OR "senior citizens").ti,ab,kf.  AND  (migrant* OR immigrant* OR refugee* OR "displaced persons" OR newcomer* OR "foreign-born").ti,ab,kf.  AND  (equit* OR fairness OR "social justice" OR "health equit*" OR disparit* OR "access to care" OR "inclusive services").ti,ab,kf.  AND  ("healthcare access" OR "health service* utilization" OR "well-being" OR "quality of life" OR "support system*" OR "culturally competent care" OR "determinants of health").ti,ab,kf.  LIMIT TO (humans AND English AND yr="2011 - 2025") | 40 |
| CINAHL with Full Text via EBSCOhost) | (("Aging" OR "Ageing" OR "Geriatrics" OR "Aging Population" OR "Older Adults" OR "Elderly" OR "Senior Citizens")  AND ("Migrant*" OR "Immigrant*" OR "Refugee*" OR "Displaced Persons" OR "Newcomer*" OR "Foreign-born")  AND ("Equit*" OR "Fairness" OR "Social Justice" OR "Health Equit*" OR "Disparit*" OR "Access to Care" OR "Inclusive Services")  AND ("Healthcare Access" OR "Health Service* Utilization" OR "Well-being" OR "Quality of Life" OR "Support System*" OR "Culturally Competent Care" OR "Determinants of Health"))  LIMIT TO: (English Language) AND (Publication Year: 2011-2025) | 115 |
| PsycINFO via EBSCOhost) | (("Aging" OR "Ageing" OR "Geriatrics" OR "Aging Population" OR "Older Adults" OR "Elderly" OR "Senior Citizens")  AND ("Migrant*" OR "Immigrant*" OR "Refugee*" OR "Displaced Persons" OR "Newcomer*" OR "Foreign-born")  AND ("Equit*" OR "Fairness" OR "Social Justice" OR "Health Equit*" OR "Disparit*" OR "Access to Care" OR "Inclusive Services")  AND ("Healthcare Access" OR "Health Service* Utilization" OR "Well-being" OR "Quality of Life" OR "Support System*" OR "Culturally Competent Care" OR "Determinants of Health"))  LIMIT TO: (English Language) AND (Publication Year: 2011-2025) | 68 |
| Scopus via Elsevier | ( TITLE-ABS-KEY ( "Aging" OR "Ageing" OR "Older Adults" OR "Elderly" OR "Senior Citizens" OR "Geriatrics" OR "Aging Population" ) AND TITLE-ABS-KEY ( "Migrant*" OR "Immigrant*" OR "Refugee*" OR "Displaced Persons" OR "Newcomer*" OR "Foreign-born" ) AND TITLE-ABS-KEY ( "Equit*" OR "Fairness" OR "Social Justice" OR "Health Equit*" OR "Disparit*" OR "Access to Care" OR "Inclusive Services" ) AND TITLE-ABS-KEY ( "Healthcare Access" OR "Health Service* Utilization" OR "Well-being" OR "Quality of Life" OR "Support System*" OR "Culturally Competent Care" OR "Determinants of Health" ) ) AND AND PUBYEAR > 2010 AND ( LIMIT-TO ( LANGUAGE , "English" ) ) | 137 |
| Web of Science via Clarivate | ALL=("Aging" OR "Ageing" OR "Older Adults" OR "Elderly" OR "Senior Citizens" OR "Geriatrics" OR "Aging Population")  AND  ALL=("Migrant*" OR "Immigrant*" OR "Refugee*" OR "Displaced Persons" OR "Newcomer*" OR "Foreign-born")  AND  ALL=("Equit*" OR "Fairness" OR "Social Justice" OR "Health Equit*" OR "Disparit*" OR "Access to Care" OR "Inclusive Services")  AND  ALL=("Healthcare Access" OR "Health Service* Utilization" OR "Well-being" OR "Quality of Life" OR "Support System*" OR "Culturally Competent Care" OR "Determinants of Health")  AND  PY=(2011-2025)  AND  LA=(English) | 162 |
| **Grand total** |  | **522** |
| **Duplicate** |  | **171** |
